# An Increased Dietary Supply of Medium-Chain Fatty Acids during Early Weaning in Rodents Prevents Excessive Fat Accumulation in Adulthood

**DOI:** 10.3390/nu9060631

**Published:** 2017-06-20

**Authors:** Bert J. M. van de Heijning, Annemarie Oosting, Diane Kegler, Eline M. van der Beek

**Affiliations:** 1 Nutricia Research, Early Life Nutrition, Utrecht, The Netherlands; annemarie.oosting@danone.com (A.O.); dianekegler@gmail.com (D.K.); eline.vanderbeek@danone.com (E.M.v.d.B.); 2 Department of Pediatrics, University Medical Center Groningen, University of Groningen, Groningen, The Netherlands

**Keywords:** early life, programming, Wistar rats, C57Bl/6j mice, body composition, infant nutrition, obesity, prevention, lactation

## Abstract

Medium-chain fatty acids (MCFA) are a directly and readily absorbed source of energy. Exposure early-in-life to increased MCFA levels might affect development and impact (lipid) metabolism later in life. We tested whether an increased MCFA intake early-in-life positively affects adult body composition and metabolic status when challenged by a western-style diet (WSD). Male offspring of C57Bl/6j mice and Wistar rats were fed a control diet (CTRL; 10 w% fat, 14% MCFA) or a medium-chain triglycerides (MCT) diet with 20% MCFA until postnatal (PN) day 42, whereupon animals were fed a WSD (10 w% fat) until PN day 98. Body composition was monitored by Dual Energy X-ray Absorptiometry (DEXA). In rats, glucose homeostasis was assessed by glucose tolerance test (GTT) and insulin tolerance test (ITT); in mice, the HOmeostasis Model Assessment of Insulin Resistance (HOMA-IR) was calculated. At autopsy on PN day 98, plasma lipid profiles, glucose, insulin, and adipokines were measured; organs and fat pads were collected and the adipocyte size distribution was analysed. Milk analysis in mice showed that the maternal MCT diet was not translated into milk, and pups were thus only exposed to high MCT levels from early weaning onward: PN day 16 until 42. Mice exposed to MCT showed 28% less fat accumulation vs. CTRL during WSD. The average adipocyte cell size, fasting plasma triglycerides (TG), and leptin levels were reduced in MCT mice. In rats, no effects were found on the adult body composition, but the adipocyte cell size distribution shifted towards smaller adipocytes. Particularly mice showed positive effects on glucose homeostasis and insulin sensitivity. Increased MCFA intake early-in-life protected against the detrimental effects of an obesogenic diet in adulthood.

## 1. Introduction

Accumulating evidence shows that exposure to environmental factors (including diet) during critical developmental periods (fetal life, infancy, early childhood), can significantly impact later-life health and metabolism [1,2]. Both over- and under-nutrition during pregnancy and infancy has been shown to result in a higher susceptibility to obesity and in poor metabolic health in adulthood [3,4,5]. Apart from nutrient quantity, nutrient quality, particularly fatty acid (FA) composition, is suggested to play a role in the nutritional programming of the adult metabolic phenotype [6,7]. Therefore, the rapidly increasing incidence of obesity [2,8] cannot solely be explained by contemporary lifestyle factors as an unbalanced diet or reduced physical activity [9]: early-life nutritional programming might also be involved.

The inclusion of medium-chain triglycerides (MCT) in the adult diet has been shown to improve insulin sensitivity, to reduce subcutaneous and visceral fat deposition, and thus to reduce body weight [10,11,12,13]. Underlying these immediate effects of MCT may be their different route of absorption in contrast to long-chain (LC) FA, i.e., >C12, medium-chain fatty acids (MCFA) (C8-12) are mainly absorbed via the portal vein, and as MCFA readily cross the mitochondrial membrane (mainly carnitine-independently), they are rapidly oxidised in liver and muscle to supply energy and body heat [14,15]. This is why high dietary MCT levels spare LCFA from oxidation, thus promoting LCFA plasma levels and their storage in adipose cells. Moreover, MCFA are almost completely oxidized and minimally stored in fat tissue. Because of their rapid and complete oxidation, MCFA are ketogenic and have (hence) a high satiating effect [15].

Given the direct effects of MCFA on fat pad weight, we speculated that an increased dietary MCFA content early in life would positively modulate metabolic settings and/or the development of metabolic organs (liver, pancreas) and white adipose tissue (WAT), thereby affecting the susceptibility to develop obesity later in life. Therefore, an increased intake of MCFA in early postnatal life would result in a lower fat mass accumulation during adulthood. 

## 2. Methods and Materials

### 2.1. Rationale

To test our hypothesis, the offspring of two rodent species, C57Bl/6j mice and Wistar rats, were used; both of which are commonly used models to study metabolic programming [16,17,18,19,20]. The experimental design, intended to expose offspring to increased MCFA levels during lactation and early post-weaning, would enable us to selectively investigate the importance of early dietary FA composition during a critical period for WAT development [21]. The early diet is discontinued before the onset of puberty, because this is considered a separate critical window of development [22,23] which is not in the scope of our research objective.

We used mice to gain insight into the programming effects on body composition over time, and functional tests on insulin sensitivity in rats (a bigger species) to assess the programming effects on the adult metabolic phenotype in more detail; adipose cell size distribution was assessed in both species.

### 2.2. Animals

All experimental animal procedures were approved by an independent ethics committee (DEC-Consult, Soest, The Netherlands), complied with the European Directive for the protection of animals used for scientific purposes, and were in line with the ARRIVE guidelines.

Mice and rats were housed at the animal facilities of Wageningen University and Research centre (WUR, Wageningen, The Netherlands) on a 12/12 h light/dark cycle (lights on at 06:00 a.m.) in a temperature- and humidity-controlled room (21 ± 2 °C and 50% ± 5%, respectively). Food and water were available *ad libitum* during the entire experimental protocol, except before blood sampling when animals were (adequately) fasted for 4 h [24,25] during the light phase (no food available, only water, from 07:30 until 11:30 a.m.). Food intake was measured per cage (i.e., per two animals) twice per week by weighing the remaining diet in the food hopper. Body weight was measured twice per week: by litter before weaning and individually afterwards.

### 2.3. Experimental Diets

All diets were semi-synthetic, consisting of AIN93G ingredients (Research Diet Services, Wijk bij Duurstede, The Netherlands), and contained 21 En% fat, 17 En% protein, and 62 En% carbohydrates. Minerals and vitamins were according to AIN93G purified diets for laboratory rodents [26]. Animals were assigned on postnatal (PN) day two to either a control (CTRL) diet which contained a vegetable oil blend (Supplementary Table S1A,B) with an FA composition similar to infant milk formula (IMF), or to a diet which contained an oil blend with ~50% extra MCFA as triglycerides (MCT). Table 1 shows the FA composition of the diets used. The diets differed in FA composition, not in the total FA content, and contained lower amounts of poly-unsaturated FA (PUFA) than standard rodent chow [25]. On PN day 42, animals were switched to a western style diet (WSD), containing 21 En% fat that consisted of 11 En% lard, 10 En% vegetable oils, and 1 g/kg cholesterol (Supplementary Table S1A,B). Compared to normal rodent chow, the WSD was low in PUFA, high in (saturated) fat and cholesterol (0.1 w%), and had a high linoleic acid/alpha linolenic acid (LA/ALA) ratio of 9.26.

### 2.4. Mouse Study

**Figure 1:**
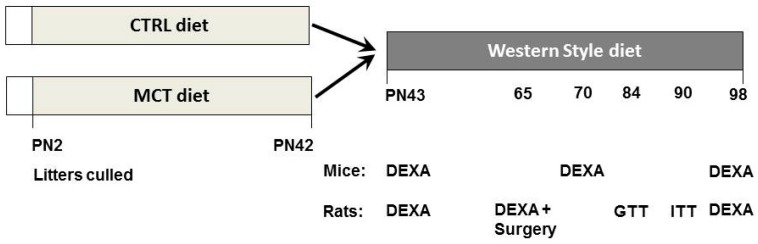
Experimental design of the rat and mouse study. Litters were randomized and culled on postnatal (PN) day two, and were exposed to a CTRL or MCT diet (*n* = 3 litters/diet). Male pups continued their respective diets after weaning (on PN day 21) until they were switched to a western style diet (WSD) on PN day 42. Body composition was monitored by dual X-ray absorptiometry (DEXA) at the indicated time points. Rats were equipped on PN day 65 with a jugular vein cannula to enable repeated stress-free blood sampling for the purpose of an intravenous glucose (GTT) and insulin (ITT) tolerance test on PN day 84 and 90, respectively.

Male and (multiparous) female C57Bl/6j mice were obtained from WUR (Wageningen, The Netherlands) and were time-mated: One male was introduced for three days in the home cage of two females. After two weeks, females were checked for pregnancy and housed individually. On PN day two, litters born within 48 h were combined and culled to a mixed-sex litter, including four males/two females, to secure the normal nursing behaviour of the dam. The male pups served as the subjects in this study and were followed into adulthood. Litters were randomly assigned to one of two experimental diets: CTRL or MCT (*n* = 3 litters/diet group). After weaning on designated PN day 21, male littermates were housed in pairs and continued their respective diets until PN day 42 (*n* = 12/diet group), a period corresponding with infancy and childhood in humans [27]. Pups were fed WSD during adolescence and adulthood until PN day 98. The body composition was measured on PN day 42, 70, and 98 by Dual Energy X-ray Absorptiometry (DEXA; PIXImus, GE Lunar, Madison, WI, USA) under general anaesthesia (isoflurane/N_2_O/O_2_). Figure 1 depicts the design of the study.

Historic pool data, as referred to previously [25], on the body weight and composition of non-challenged chow-fed male mice at PN day 98 were used to determine the effects of the WSD challenge; body weights on PN day 42 were not different from the intervention groups.

In a separate study, and published partly already elsewhere [28], we obtained mid-lactation milk samples from dams on PN day 12 (*n* = 5/diet group) to assess to what extent the diet of the dams affected the FA composition of their milk. Milk was withdrawn using an adjusted human lactation pump 10 min after administering oxytocin (0.3 mL subcutaneously, 1 IU/mL; Eurovet, Bladel, The Netherlands) to lactating dams isolated for 3 h from their litter. See elsewhere for details [28]. Additionally, the FA composition of the erythrocyte membranes of the male pups was assessed at weaning (PN day 21) as a proxy index for dietary impact [29].

### 2.5. Rat Study

In a similar way as described for mice, male and primiparous female Wistar Unilever (WU) rats (HsD/Cpb-WU, Harlan laboratories, Horst, The Netherlands) were time-mated, and the litters (now four male and four female pups) were assigned on PN day two to either a CTRL or MCT diet (*n* = three litters/diet group). Upon weaning at PN day 21, male littermates (*n* = 12/diet group) were pair-housed and continued on their respective diets until PN day 42, and were subsequently changed to WSD until PN day 98.

On PN day 42, blood samples were taken by orbital puncture under general anaesthesia (isoflurane/N_2_O/O_2_) after 4 h of fasting. The body composition was measured on PN day 42, 65, and 98 by DEXA (Discovery A, Hologic Inc., Bedford, MA, USA) under general anaesthesia.

As in the mouse study, historic data (referred to in [25]) on the body weight and composition of non-challenged chow-fed male Wistar rats were used to assess WSD challenge effects.

On PN day 65, the rats were anesthetised and equipped with a silicone catheter in the right jugular vein to allow repeated stress-free blood sampling in conscious animals. After full recovery, on PN day 84, an intravenous glucose tolerance test (GTT) was performed in the now individually housed rats. Briefly, blood samples were taken at −10, 2, 4, 6, 8, 10, 15, 20, and 30 min after the intravenous administration of 500 mg/kg glucose (500 mg/mL saline). Next, on PN day 90, an intravenous insulin tolerance test (ITT) was performed during which 0.5 U/kg insulin (I-5500, Sigma Aldrich Chemie, Zwijndrecht, The Netherlands; 29 U/mg, 27.59 µg/mL saline) was intravenous administered, whereupon blood samples were taken at −10, 2, 4, 6, 8, 10, 15, 20, 30, and 60 min. GTT and ITT samples were collected in K_3_EDTA-coated microtubes (Greiner Bio-one, Alphen aan den Rijn, The Netherlands) and centrifuged at 1350× *g* during 15 min at 4 °C (Biofuge fresco, Heraeus, Hanau, Germany). Plasma was isolated and stored at −80 °C until analysis.

### 2.6. Termination and Dissection

On PN day 98, blood samples were taken from both mice and rats by cardiac puncture under terminal anaesthesia (isoflurane/N_2_O/O_2_), after which the animals were sacrificed for dissection. Blood was collected in K_3_EDTA-coated microtubes. Plasma was obtained by centrifugation at 1350× *g* for 15 min at 4 °C and stored at −80 °C. The liver, pancreas, epididymal (EPI), retroperitoneal (RP), perirenal (PR), and inguinal (ING) fat depots were collected and weighed. 

### 2.7. Cytological and Biochemical Analyses of Mouse and Rat WAT

The adipocyte size distribution, DNA, and TG content in mouse EPI were determined in the fixated tissue collected on PN day 98, as previously described [24]. A total of 12–24 microscopic pictures of the fat pads of each animal were analysed. In rats, the adipocyte size distribution was determined in fresh ING and RP depots according to the optical method of DiGirolamo [30]. Depots were cut into ~1 mm^3^ pieces using a McIlwain tissue chopper (Mickle laboratory engineering Co. Ltd, Gomshall, UK) and incubated at 37 °C for 60 min in a shaking water bath (60–80 strokes/min) in gassed (95% O_2_/5% CO_2_) Krebs Ringer bicarbonate buffer (KRB) at pH 7.4 containing 2 mg/mL collagenase type II (Gibco for Invitrogen, California, CA, USA). The digested tissue was filtered through a cell strainer with a 250 µm nylon mesh. The freed adipocytes in the filtrate became afloat, and were collected and washed three times with KRB buffer. The infranatant was filtered through a cell strainer with a 40 μm nylon mesh and the freed cells were added to the collected adipocytes.

70 µL Aliquots of the adipocyte suspension were placed on a slide and covered with a cover slip. Images were captured using an Axioplan 2 Zeiss microscope (Carl Zeiss, Weesp, The Netherlands) and a Sony DXC-950P video camera (Sony, Badhoevedorp, The Netherlands) at 100× magnification. Six representative sections per slide and six slides per depot per rat were used to obtain at least 400 cells to assess the mean adipocyte volume and size distribution per animal with analySIS software (Soft imaging system, Münster, Germany).

The WAT lipid content was determined according to the method of Folch [31] using extraction with dichloromethane: methanol (2:1 *v*/*v*).

### 2.8. Plasma Analyses

Fasting plasma total cholesterol (TC; cholesterol liquicolor CHOD-PAP, Instruchemie, Delfzijl, The Netherlands), triglycerides (TG; GPO trinder method, Sigma Aldrich, Zwijndrecht, The Netherlands), free fatty acids (FFA; NEFA-C method, Wako Chemicals, Neuss, Germany), and glucose (GOD-PAP method, Roche diagnostics, Almere, The Netherlands) were measured colorimetrically and analysed with a microplate imaging system (Bio-Rad Laboratories Inc.^®^, Hercules, CA, USA). The total adiponectin in mice was determined using an ELISA kit (Linco Research, Billerica, MA, USA). Plasma insulin, leptin, monocyte chemoattractant protein 1 (MCP-1), total plasminogen activator inhibitor-1 (tPAI-1), interleukin 6 (IL-6), tumor necrosis factor α (TNFα), and resistin in mouse plasma were measured simultaneously using a mouse serum adipokine lincoplex kit (Linco Research). Samples, standards, and quality control were prepared according to manufacturers’ protocol and fluorescence was measured using a Bio-Plex™ 200 Luminex instrument (Bio-Rad Laboratories, Hercules, CA, USA). As an indirect measure of insulin sensitivity [32,33], the homeostasis model assessment of insulin resistance (HOMA-IR) was calculated using the fasting plasma levels of glucose and insulin: (glucose (mM) × insulin (pM)/22.5). Rat insulin was analysed by ELISA (Rat Insulin ELISA, DRG Diagnostics International Inc., Springfield Township, NJ, USA), according to the manufacturer’s protocol.

### 2.9. Statistical Analyses

Statistical analyses were performed using SPSS 19.0 (SPSS Benelux, Gorinchem, The Netherlands). The effects of diets on erythrocyte FA composition, body weight (BW), food intake, body composition, organ weight, plasma parameters, glucose tolerance, insulin sensitivity, adipocyte size distribution, and mean adipocyte size were analysed using mixed effects regression models, with an early diet as the fixed effect. In all regression models, the correlation among animals within the litter was accounted for by their sharing of a common random effect. Pair wise comparisons were adjusted for multiple comparisons, using the post-hoc LSD (least square difference) test. A student’s *T*-test was used to analyse the effects of diet on the milk FA composition. Differences were considered significant when *p* < 0.05. Data are shown as means and standard deviation (SD).

## 3. Results

### 3.1. Effects of Early Diet on Milk and Pup Erythrocyte FA Composition

Confirming our previous data, an analysis of the milk at PN day 12 (Supplementary Table S2) showed that the MCT diet was not translated into the milk FA composition, which was similar to the CTRL group. Furthermore, the FA composition of the erythrocyte membranes of pups at weaning (PN day 21) did not differ between the two groups and confirmed the absence of changes directly or indirectly due to the milk FA composition (Supplementary Table S2). Hence, in contrast to the experimental design, pups in the MCT group were exposed to a high MCT intake only from early weaning onward, i.e., from PN day 16 until 42.

### 3.2. Direct Effects of Early Diet on Growth, Body Composition, and Food Intake

**Figure 2:**
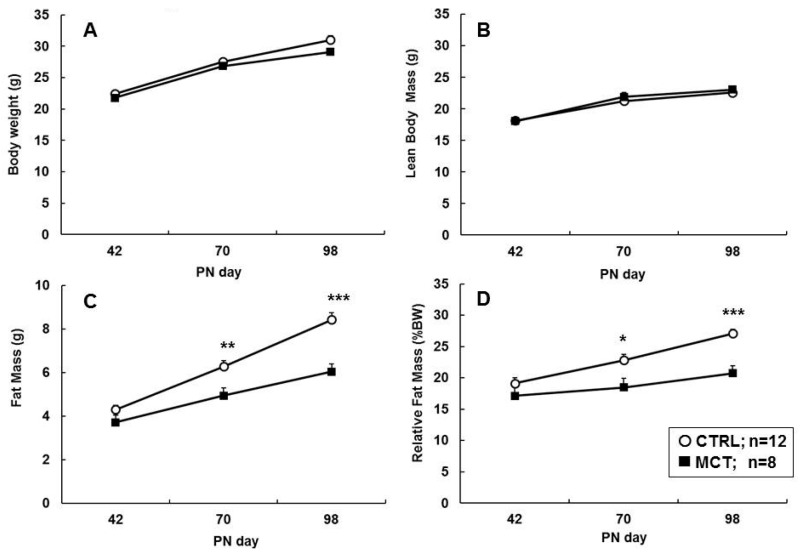
Mouse study Development of body weight (BW), lean body mass (LBM), fat mass (FM), and relative fat mass (%BW) during the WSD challenge (PN 43–98) of male mice (panels (**A**–**D**), respectively) fed a CTRL (*n* = 12) or MCT diet (*n* = 8) in early life (PN day 2–42). Values are means ± SD; * *p* < 0.05; ** *p* < 0.01; *** *p* < 0.001 vs. CTRL.

**Figure 3:**
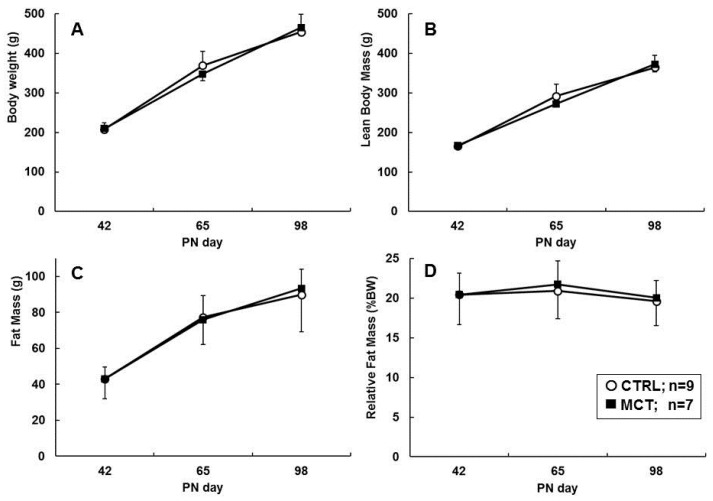
Rat study Development of body weight (BW), lean body mass (LBM), fat mass (FM), and relative fat mass (%BW) during the WSD challenge (PN 43–98) of male **rats** (panels (**A**–**D**), respectively) fed a CTRL (*n* = 9) or MCT diet (*n* = 7) in early life (PN day 2–42). Values are means ± SD.

The body weight (BW) at PN day 42 did not differ between mice and rats fed a CTRL or MCT diet, was and the lean body mass (LBM), fat mass (FM), or relative FM (%BW) was not different between diet groups directly after the dietary intervention (Figure 2 and Figure 3). Additionally, the food intake at PN day 42 was comparable between CTRL and MCT groups in both species (Table 2).

### 3.3. Effects of Early Diet on Food Intake and Body Composition during WSD Challenge

The average daily WSD intake (Table 2) was similar in animals postnatally fed MCT compared to CTRL. On PN day 98, CTRL mice had accrued 18% BW extra (i.e., 5.3 g BW, of which 84% adipose tissue, 4.4 g) due to the WSD challenge compared to age-matched mice reared from weaning onward on a normal AIN-diet (historic data). This means that the WSD challenge doubled (+52%) the FM at PN day 98, which represents an increase of about 12 w% in relative FM. 

Although the BW gain did not differ significantly between CTRL and MCT mice during WSD exposure (Figure 2A), the absolute FM was 28% lower and the relative FM was 23% lower in MCT than CTRL mice at PN day 98 (Figure 2C,D, respectively). LBM development was similar in both groups (Figure 2B). In accordance with a lower total FM, the weights of the WAT depots collected at dissection were reduced: EPI and ING pad weights were lower, and also RP depots tended to be smaller in MCT than CTRL mice (Table 3). The brain, kidney, and thymus weights were similar between CTRL and MCT. The pancreas weight was numerically higher in the MCT group; this difference reached marginal statistical significance (*p* = 0.028, one tailed). The MCT liver weights tended to be smaller (*p* = 0.052, one tailed) than CTRL, but the liver TG content was significantly lower in MCT animals. The relative organ weights (expressed as % BW) showed similar results.

Rats fed the WSD challenge showed the same BW gain and/or body composition, and no differences were observed compared to the age-matched rats reared on normal AIN (historic data, not shown). Both early diet interventions yielded a similar body weight gain, LBM accrual, FM, and relative FM gain due to the WSD challenge in both CTRL and MCT rats (Figure 3A–D, respectively). 

Rats responded differently to the WSD challenge than mice, and FM gain in all rats kept pace over time with the increasing LBM, which resulted in a stable relative FM during PN day 42 to 98 (Figure 3D). Accordingly, comparable liver, pancreas, and WAT depot weights were found at PN day 98 in both CTRL and MCT rats (Table 3).

### 3.4. Effects of Early Diet on Adipocyte Size Distribution after WSD Challenge

**Figure 4:**
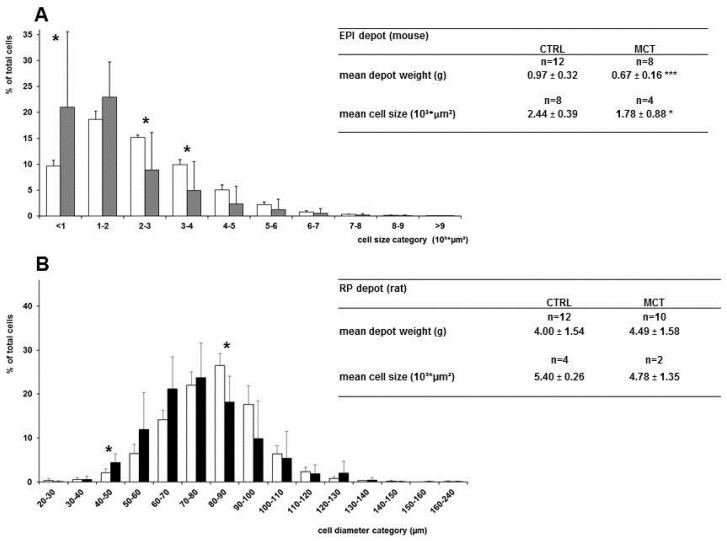
Panel (**A**) Frequency distribution of EPI adipocyte cell size in CTRL (*n* = 8; white bars) and MCT (*n* = 4; grey bars) mice on PN day 98; Panel (**B**) Frequency distribution of RP adipocyte cell size in CTRL (*n* = 4; white bars) and MCT (*n* = 2; black bars) rats on PN day 98. The tables show the mean depot weight and cell size. Values are means ± SD; * *p* < 0.05; *** *p* < 0.001 vs. CTRL; EPI, epididymal fat; RP, retroperitoneal fat.

In mice, the adipocyte size distribution of the EPI fat pad is shown in Figure 4A: MCT mice had fewer cells with a cell surface area of 2–4 (10^3^ µm^2^) compared to CTRL mice, which is also reflected in their mean adipocyte cell size (*p* = 0.049, one tailed), as mentioned in Figure 4A. This smaller cellularity of the EPI fat pad resulted in a higher DNA content per mg WAT (Table 4). Next, as the fat pad TG content (mg/mg) was similar between groups, it follows that adipocytes in the MCT group contain a lower TG load per cell (TG/DNA), again in line with a smaller cellularity compared to CTRL (Table 4).

In rats, fat pad cellularity analysis was carried out somewhat differently in the 30–36 fat pad tissue blocks (~1 mm^3^) of each animal; Figure 4B summarises the results for the RP fat depots (Supplemental Figure S1 shows the rat ING data). Although fat pad weights and the mean cell size did not differ between the two diet groups (Table 3 and Figure 4), the MCT early diet yielded a moderate shift to the left, towards smaller cells in both the RP and ING fat pads, which reached statistical significance in only a few cell diameter categories. 

### 3.5. Effects of Early Diet on Metabolic Plasma Parameters after WSD Challenge

The fasting plasma cholesterol (TC) was lower in MCT than CTRL mice. In contrast, TG and FFA were similar in both groups, as were most plasma adipokines (Table 4), except for leptin levels, which were lower in MCT than CTRL mice, entirely in line with the lower adipocity. Glucose homeostasis tended (*p* < 0.1) to be better in the MCT diet group: lower fasting levels of glucose and insulin, and hence a lower HOMA-IR value, were observed.

**Figure 5:**
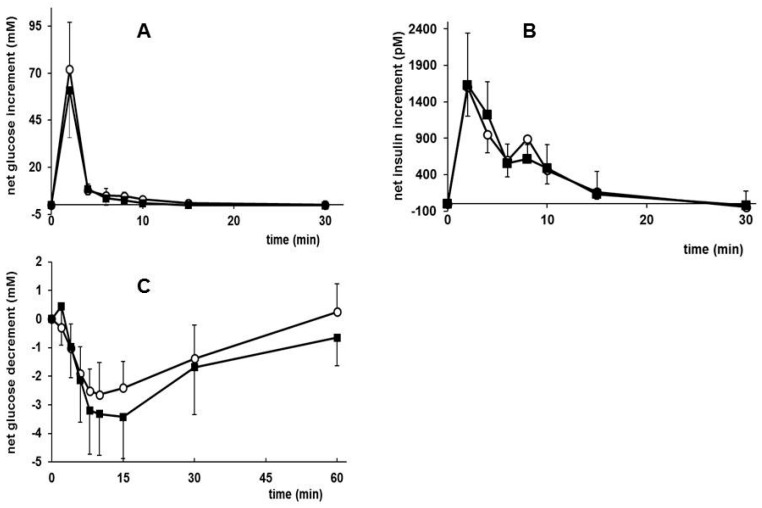
Results of the intravenous GTT (panel (**A**) and (**B**)) and intravenous ITT (panel (**C**)) carried out on PN day 84 and 90, respectively, in male rats fed a CTRL (*n* = 11–12) or MCT diet (*n* = 7–8) in early life (PN day 2–42). Values are means ± SD.

In rats, only the fasting plasma glucose, but not insulin or HOMA-IR, were lower in the MCT diet group directly after dietary intervention at PN day 42 (Supplementary Table S3). No differences were observed in glycemic control after the WSD challenge (at PN day 98): both groups had a similar glucose homeostasis, i.e., increased fasting glucose and insulin levels. At PN day 84, an intravenous GTT was carried out in the cannulated rats, and the results (in Figure 5A) show that MCT rats are equally as glucose tolerant as CTRL animals, although the MCT group showed a trend towards a lower area under the net glucose curve (iAUC: incremental Area Under the Curve, iAUC; *p* = 0.061, one tailed) compared to CTRL rats (Supplementary Table S3). Insulin responded equally well in both groups to the intravenous administered glucose (Figure 5B). The absolute and net maximal increase of both glucose and insulin did not differ between groups (Supplemental Table S3).

In line with this, the response to the intravenous ITT carried out at PN day 90 revealed that MCT rats tended to be more insulin-sensitive than CTRL. The net plasma glucose decrement (*p* = 0.066, one tailed), but not the dAUC: decremental Area Under the Curve in response to insulin (*p* = 0.12), tended to be bigger in MCT than CTRL rats (Figure 5C and Supplementary Table S3).

## 4. Discussion

In accordance with our hypothesis, we clearly showed that an increased postnatal MCFA intake protects against excessive fat accumulation and induces a beneficial metabolic phenotype in adulthood. In addition, our study provides specific support for the importance of the post-lactational, early weaning period as part of the critical time window in metabolic development, as it was shown that the increased MCT intake of the lactating dams was not translated into their milk composition, confirming previous observations in this respect [28]. So, in contrast to the aimed experimental design (Figure 1), the dietary intervention fully and effectively reached the offspring from PN day 21 onward, but increased from around PN day 15 onward when the natural weaning process started and the pups started to eat from the diet themselves.

The erythrocyte membrane FA composition data collected on PN day 21 indeed confirm this view and showed no differences between diet groups. We assessed the milk and erythrocyte membrane FA composition only in mice and assumed that the same would be true for rats.

Both the duration and timing of a nutritional intervention impact on the particular type of mechanism invoked and/or its (programming) effects were elicited [1,34]. Most programming studies therefore span gestation and/or lactation; interventions during the postnatal period and in particular during early weaning are scarce. We previously demonstrated that a dietary lipid manipulation between PN days 15 and 42 effectively programmed the adult body composition [35], and also, our present study confirms a later start of dietary exposure to be sufficient to yield programming effects. These data confirm the notion that early weaning is part of the critical period of development that affects lifelong metabolic health outcomes.

How the early life MCFA exposure exerted its effect is as often hard to disentangle [34] and remains unclear. The 24-h food intake, but not the intake patterns or circadian rhythm, was monitored for both groups and species, but no differences (Table 2) were found on PN day 42 and 84, precluding a possible (direct) cause driving the differences in BW and body composition found at PN day 98. Yet, this does not exclude a changed (hypothalamic) regulation of satiety and energy expenditure, as shown to hold for the impact of *n*-3 and *n*-6 PUFA levels on growth and body composition [36,37].

Previous studies showed the programming effects of an increased MCFA intake (~53 FA%) during pregnancy and lactation in rats [16]: An increased insulin output, but a similar plasma glucose profile was observed, indicating a decreased insulin sensitivity. The latter was not concluded by the authors, who concentrated more on the morphological effects found in the pancreas (amongst others decreased islet volumes and numbers). We found only marginal effects on the pancreas size, improved insulin sensitivity in mice, but no effects on functionality (i.e., glucose clearance) in rats despite the mild WSD challenge. More recently, MCFA programming was reported during pregnancy only, followed by a high-fat diet (HFD; 35 En%) challenge [38]. In line with our data, Dong and co-workers found a prevention of later-life obesity in the offspring, which was found to be accompanied (or caused) by a persistent down-regulation of several metabolism-related enzymes. The authors claimed that the altered gene expression only became manifest upon the HFD challenge. As we did not assess gene expression, we do not know if these changes are also present or underlie the effects observed in the present study. In the case of an MCFA intervention during pregnancy, the offspring is only indirectly exposed via maternal blood, whereas in our case, the offspring was exposed directly to MCFA. An analysis of maternal blood composition that could confirm that maternal MCFA-enriched diet reached the offspring was not performed in the Dong study.

MCFA are not known to affect adipogenesis directly, which drives WAT development, but could have affected adipogenesis indirectly. Adipogenesis entails both the proliferation and differentiation of pre-adipocytes to mature adipocytes, thus yielding a certain lipid storage capacity. The adipocyte number is established in humans during childhood and adolescence [39] and also in rodents, i.e., around 60–80 days of age [21,40,41], well after the dietary intervention and during the WSD exposure period in our current study. Adipocyte size depends on dietary intake and hence can increase throughout life. A higher dietary MCT intake was shown to promote lipogenesis [42], probably by sparing both glucose and LCFA oxidation, thus facilitating fat accumulation and enabling *n*-3 and *n*-6 PUFA to differentially modulate pre-adipocytes proliferation and differentiation [7,43,44]. Indeed, dietary *n*-3 PUFA during pregnancy and/or lactation may program towards a reduced fat mass in animal [7,20,45,46] and human subjects [47,48,49]. In contrast, high levels of *n*-6 PUFA are associated with enhanced adipogenesis in humans and rodents [50]. We previously showed that exposing mouse pups to increased levels of *n*-3 PUFA [24] or to decreased levels of *n*-6 PUFA [24,25] in their early postnatal diet protected against the detrimental effects of an obesogenic diet during adulthood on metabolic health and fat mass accumulation. 

The two postnatal diets used had a similar SFA, MUFA, and PUFA content, equal LA/ALA and *n*-6/*n*-3 ratio, and differed only in the MCT moiety (as part of the SFA fraction). We did not determine the plasma FA composition in the programmed animals (PN day 42), and thus cannot rule out a possible effect on the PUFA levels or balance. However, no differences were observed in the FA composition of erythrocytes from the offspring obtained on PN day 21.

The almost 30% reduced adult fat mass accumulation and smaller mean adipocyte size in the EPI depot in the MCT mouse group despite a similar WSD intake, suggests a reduced lipid-loading of adipocytes compared to the CTRL group. A different energy expenditure and/or lipid trafficking in the MCT group due to a differently programmed metabolic homeostasis might explain our results, although the methodology used did not allow the cell numbers to be reliably estimated.

In rats, we observed no effect on the body composition or fat depot weights, which is likely explained by the modest WSD challenge during adulthood. This WSD diet did not result in any increased fat mass development compared to a standard chow (historic data group). In contrast, in mice, the WSD challenge yielded almost a doubled fat accumulation in the CTRL group compared to AIN chow mice (historical data group). In rats, a marginal shift in adipocyte size distribution was observed, which was more pronounced in the RP (visceral) than in the ING (subcutaneous) depot; in both fat depots, the mean cell size was not significantly altered. Although the number of depots analysed were small and the adipocyte numbers were unknown, an overall shift to the left, towards smaller sized cells, was observed in the MCT-group, assuming a lower degree of lipid accumulation in the MCT vs. CTRL rats. The WSD challenge used (10 w%, 21 En% fat) should be considered mild, particularly for rats, compared to the high fat diets (>45 En% fat) applied elsewhere [51], but also compared to human western dietary fat intakes on which the WSD design is based [52]. Proof that an increased postnatal MCFA exposure may prevent excessive fat accumulation in rats might require a highly obesogenic diet and/or a longer WSD exposure in this species. A comparison of the mice and rat data (Figure 2 vs. Figure 3) indicates that in rats, the LBM gain continues to increase, as does FM, during the WSD period (PN day 42 to 98), which results in a flat and stable %FM in contrast to mice and may explain the species differences observed. The reduced adult fat mass in mice was accompanied by lower plasma leptin levels, but an otherwise similar metabolic phenotype, although the HOMA-IR value was improved. In rats, in line with the phenotypic effects, glucose tolerance and insulin sensitivity were only marginally affected. Again, the WSD challenge might have been too mild for this species.

The effects of increasing the dietary MCFA intake during early development are scarcely studied [16,38]. In contrast, observational and intervention studies in adults and animals have suggested immediate beneficial effects of MCT on metabolic health in adulthood: Substitution with MCFA improved the metabolic status in obese adult subjects [12,13,14,15].

Human milk fat consists of about 8 %FA of MCFA [53], and may program the developing metabolism and WAT development of suckling newborns, as observed in our study. MCFA are added in abundance (up to 50 %FA) to infant formula, in particular to products for pre-term born infants. Its addition is mainly because of the metabolic features, i.e., being a readily available source of energy [10,42]. To this end, often specific MCT-rich oils are used (e.g., coconut or mygliol oil), but also adding cow’s milk fat (or butter oil) as an ingredient markedly increases the MCFA content of a formula. Apart from benefiting from the metabolic advantages of MCFA, both breast- and formula-fed infants might also have their developing metabolism and adipose tissue positively programmed due to the MCFA intake [42,54]. Only short-term beneficial effects of added MCFA are known and long-term consequences of high MCFA intakes have not been studied. Our mouse model indicated that exposure to a 50% increased MCFA level (effecting in a 20 %FA content) early in life protected against excessive fat accumulation in adulthood. These results merit further investigation using human observational and intervention study settings.

## 5. Conclusions

An increased MCFA intake in early life programmed resistance to an obesogenic diet during adolescence and adulthood. The present results underline the importance of the early life dietary lipid composition to support and safeguard metabolic development and health later in life.

## Figures and Tables

**Table 1:** Fatty acid (FA) composition of the experimental diets (as measured).

(%FA)		Early Diet		WSD
		CTRL ^1^	MCT	
C-8:0	Caprylic acid	1.65	15.20	1.06
C-10:0	Capric acid	1.52	3.54	0.87
C-12:0	Lauric acid	10.83	1.59	6.02
C-14:0	Myristic acid	4.45	1.13	4.17
C-16:0	Palmitic acid	18.73	14.67	23.37
C-16:1 *n*-7	Palmitoleic acid	0.15	0.15	1.2
C-18:0	Stearic acid	3.61	2.84	13.66
C-18:1 *n*-9	Oleic acid	38.16	39.84	36.22
C-18:2 *n*-6	Linoleic acid (LA)	15.26	15.15	8.80
C-18:3 *n*-3	Alpha linolenic acid (ALA)	2.96	2.97	1.67
C-20:0	Arachidic acid	0.38	0.37	0.28
C-20:1 *n*-9	Eicosaenoic acid	0.43	0.48	0.27
C-20:4 *n*-6	Arachidonic acid	0	0	0
C-20:5 *n*-3	Eicosapentaenoic acid (EPA)	0	0	0
C-22:0	Behenic acid	0.32	0.32	0.09
C-22:1 *n*-9	Erucic acid	0	0	0
C-22:5 *n*-3	Docosapentaenoic acid	0	0	0
Σ MCFA (C8:C12)		14.00	20.34	7.95
Σ SFA %		41.58	39.79	50.09
Σ MUFA %		38.74	40.51	37.70
Σ PUFA %		18.22	18.20	10.47
Σ *n*-6		15.26	15.23	8.80
Σ *n*-3		2.96	2.97	1.67
*n*-6/*n*-3 ratio		5.16	5.12	5.26
LA/ALA ratio		5.16	5.10	5.26

^1^ CTRL, control diet; MCT, medium-chain triglyceride diet; WSD, western-style diet; MCFA, medium-chain FA; SFA, saturated FA; MUFA, mono-unsaturated FA; PUFA, poly-unsaturated FA.

**Table 2:** Mean food intake (g/day) during early diet (on PN day 42) and during WSD intervention (on PN day 84) ^1^.

(g/Day)	Mice		Rats	
	CTRL	MCT	CTRL	MCT
Early diet	3.18 ± 0.26 (*n* = 6)	3.15 ± 0.40 (*n* = 4)	21.6 ± 4.5 (*n* = 3)	19.1 ± 0.8 (*n* = 3)
WSD	2.98 ± 0.35 (*n* = 6)	3.05 ± 0.51 (*n* = 4)	21.1 ± 4.8 (*n* = 12)	22.9 ± 2.1 (*n* = 10)

^1^ Values are means ± SD. CTRL and MCT diets were fed from PN day 2–42, WSD was fed from PN day 43–98.

**Table 3:** Average weight of fat depots and organs at PN 98 ^1^.

		Mouse		Rat	
		CTRL	MCT	CTRL	MCT
Fat depots	EPI (g)	0.97 ± 0.32	0.61 ± 0.17 ***	6.47 ± 1.78	6.75 ± 1.95
	PR (g)	0.11 ± 0.04	0.09 ± 0.05 **#**	1.00 ± 0.29	1.18 ± 0.15
	RP (g)	-	-	4.00 ± 1.54	4.49 ± 1.58
	ING (g)	0.64 ± 0.15	0.33 ± 0.15 ***	5.72 ± 1.75	5.94 ± 1.88
Liver (g)		1.32 ± 0.21	1.18 ± 0.20 ^#^	15.72 ± 2.87	15.42 ± 2.12
TG (w%)		13.87 ± 7.05	7.13 ± 3.53 **	-	-
mg TG/mg protein		0.30 ± 0.16	0.18 ± 0.08 **	-	-
Pancreas (mg)		92 ± 51	142 ± 14 *	1178 ± 231	1200 ± 166
Brain (mg)		452 ± 13	454 ± 18	1999 ± 122	1990 ± 79
Kidneys (mg)		350 ± 31	337 ± 125	-	-
Thymus (mg)		56 ± 6	48 ± 7	-	-

^1^ Values are means ± SD; # *p* = 0.06; * *p* < 0.05; ** *p* < 0.01; *** *p* < 0.001 vs. CTRL. CTRL and MCT diets were fed from PN day 2–42, and WSD was fed from PN day 43–98. Mice: *n* = 12 for CTRL, *n* = 8 for MCT, *n* = 4 for ING, pancreas and thymus data; RP depots were not collected. Rats: *n* = 12 for CTRL, *n* = 10 for MCT; kidneys nor thymuses were collected. EPI, epididymal; PR, perirenal; RP, retroperitoneal; ING, inguinal fat depot.

**Table 4:** Fasting plasma parameters and EPI fat depot traits at PN day 98 in mice ^1^.

			CTRL	MCT
Plasma	Adipokines	Adiponectin (mg/L)	8.64 ± 0.64	8.67 ± 1.09
		Leptin (µg/L)	4.97 ± 2.52	1.89 ± 1.01 **
		Resistin (µg/L)	4.06 ± 0.83	4.00 ± 1.24
		MCP-1 (ng/L)	9.56 ± 4.24	10.26 ± 6.81
		tPAI-1 (µg/L)	1.84 ± 0.56	2.19 ± 0.36
		TNFα (ng/L)	4.26 ± 0.46	4.60 ± 0.33
		IL-6 (ng/L)	6.36 ± 2.74	7.78 ± 5.78
	Insulin sensitivity	Glucose (mM)	16.51 ± 2.35	13.66 ± 5.07 ^#^
		Insulin (pM)	114.62 ± 87.27	64.32 ± 38.03 ^#^
		HOMA-IR	89.11 ± 75.79	41.72 ± 26.34 ^#^
	Lipids	TG (mg/L)	2.40 ± 0.70	2.60 ± 0.95
		TC (mg/L)	1262 ± 183	1017 ± 355 *
		FFA (mM)	0.50 ± 0.19	0.44 ± 0.09
EPI Fat Depot		DNA (ng/mg WAT)	32.3 ± 8.9	45.1 ± 19.4 *
		TG (mg/mg WAT)	0.84 ± 0.9	0.83 ± 0.9
		TG/DNA	28.7 ± 10.4	20.5 ± 6.2 *
		Total DNA (µg)	28.9 ± 4.1	25.8 ± 4.4 ^#^

^1^ Values are means ± SD, *n* = 8 per group. ^#^ *p* < 0.1; * *p* < 0.05; ** *p* < 0.01 vs. CTRL. CTRL and MCT diets were fed from PN day 2–42, WSD was fed from PN day 43–98. EPI, epididymal; MCP-1, Monocyte Chemoattractant Protein 1; tPAI-1, total Plasminogen Activator Inhibitor-1; TNFα, Tumor Necrosis Factor α; IL-6, interleukin-6; HOMA-IR, HOmeostasis Model Assessment of Insulin Resistance (=(Glucose × Insulin)/22.5); TG, triglycerides; TC, total cholesterol; FFA, free fatty acids; EPI, epididymal.

## Supplementary Materials

The following are available online at www.mdpi.com/2072-6643/9/6/631/s1, Figure S1: Frequency distribution of ING depot adipocyte cell size in CTRL (n = 4; white bars) and MCT (n = 3; black bars) rats on PN day 98. The table shows mean ING depot weight and cell size. Values are means ± SD; ING, inguinal fat, Table S1: Composition of the experimental diets, Table S2: Mouse milk FA composition at PN day 12 and male pup erythrocyte FA composition at weaning on PN day 21, Table S3: Glucose and insulin dynamics in fasting state and during intravenous glucose and insulin tolerance tests in rats.
