# Supplementary material for: An Increased Dietary Supply of Medium-Chain Fatty Acids during Early Weaning in Rodents Prevents Excessive Fat Accumulation in Adulthood
Source: Nutrients. 2017 Jun 20;9(6):631. doi: 10.3390/nu9060631 (PMC5490610; doi:10.3390/nu9060631)
Supplement: Supplementary file 1 [file nutrients-09-00631-s001.zip › nutrients-200574-SUPPLE.pdf]

Supplementary Figure S1

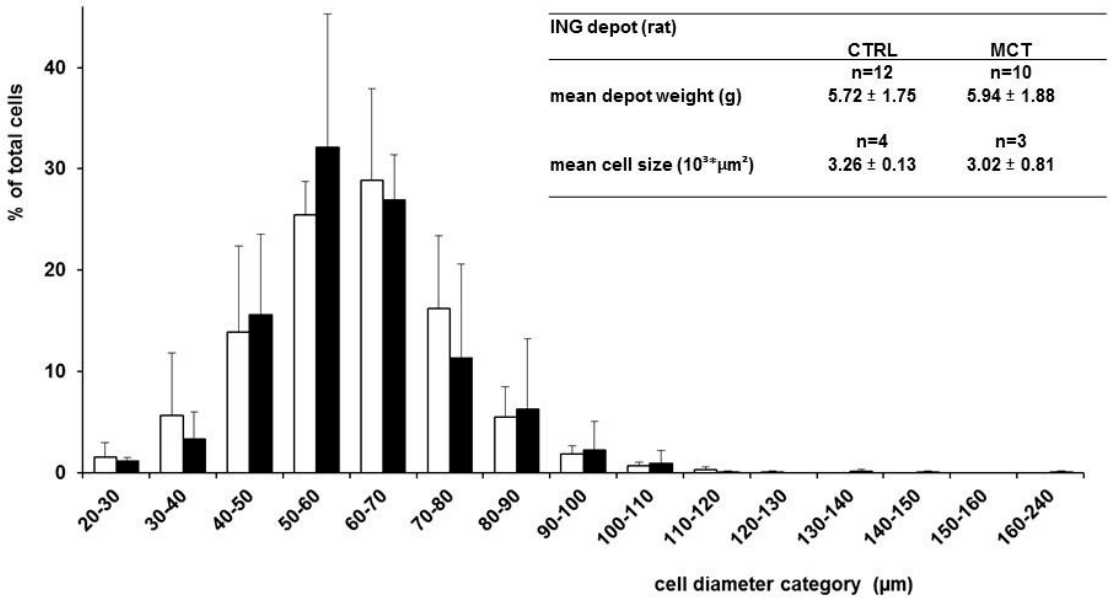

Figure S1. Frequency distribution of ING depot adipocyte cell size in CTRL (*n* = 4; white bars) and MCT (*n* = 3; black bars) rats on PN day 98. The table shows mean ING depot weight and cell size. Values are means ± SD; ING, inguinal fat.

Supplementary Table S1. Composition of the experimental diets

**A: Ingredients**	**Early diet**		**WSD**
**(g/kg)**	**CTRL**	**MCT**	
Protein (20 w%)			
Sodium caseinate	200	200	200
Carbohydrates (60 w%)			
Corn starch	450	450	450
Maltodextrin (DE19)	150	150	150
Fats (10 w%)			
Oil blend (see Table 1B and 1C)	100	100	50
Lard	0	0	50
Cholesterol	0	0	1
Fiber (5 w%)			
Cellulose (Vitacel L 600-20)	50	50	50
Other (5 w%)			
Mineral mix	35	35	35
Vitamin mix	10	10	10
Choline bitartrate	2.5	2.5	2.5
L-cysteine	3	3	3
Tertiary-butylhydroquinone	0.014	0.014	0.014

**B: Oil blend composition used**	**Early diet**		**WSD**
**(g/100 g)**	**CTRL**	**MCT**	
Rapeseed oil (Canola)	30	30	15
Sunflower HO oil	8	8	4
Trisun80 oil	6	11	3
Coconut oil	24	3	12
Palm oil	32	27	16
MCT mygliol oil	0	21	1
Lard	0	0	50
Cholesterol	0	0	0.1

Supplementary Table S2. Mouse milk FA composition at PN day 12 and male pup erythrocyte FA composition at weaning on PN day 21

**(%FA)**	**Milk**		**Erythrocyte membrane**	
	CTRL	MCT	CTRL	MCT
8:0	0.09 ± 0.06	0.13 ± 0.10	0	0
10:0	5.44 ± 0.62	5.09 ± 0.75	0	0
12:0	12.02 ± 0.76	9.98 ± 1.19	0.10 ± 0.03	0.02 ± 0.01
14:0	12.16 ± 0.87	11.21 ± 0.78	0.67 ± 0.11	0.43 ± 0.06
16:0	23.84 ± 0.85	24.55 ± 0.81	32.14 ± 0.43	31.99 ± 0.53
18:0	1.93 ± 0.35	1.96 ± 0.12	0.13 ± 0.02	0.14 ± 0.02
18:1*n*-9 (OA)	27.78 ± 1.06	29.65 ± 2.02	16.15 ± 0.33	16.64 ± 0.46
18:2*n*-6 (LA)	6.17 ± 0.34	6.26 ± 0.66	7.94 ± 0.46	7.38 ± 0.32
18:3*n*-3 (ALA)	0.61 ± 0.06	0.60 ± 0.04	0.21 ± 0.04	0.17 ± 0.02
20:4*n*-6 (AA)	0.52 ± 0.06	0.55 ± 0.08	14.02 ± 0.59	13.70 ± 0.29
20:5*n*-3 (EPA)	0.13 ± 0.05	0.14 ± 0.03	0.47 ± 0.03	0.48 ± 0.03
22:6*n*-3 (DHA)	0.20 ± 0.01	0.20 ± 0.02	6.13 ± 0.17	6.11 ± 0.20
MCFA (C8:C12)	17.56 ± 1.27	15.21 ± 1.91	0.10 ± 0.03	0.02 ± 0.01
SFA	55.56 ± 1.59	53.07 ± 2.69	42.29 ± 0.31	42.25 ± 0.53
MUFA	34.87 ± 1.54	37.27 ± 2.13	21.25 ± 0.26	22.27 ± 0.61
PUFA	9.47 ± 0.34	9.66 ± 0.69	34.50 ± 0.30	33.56 ± 0.36
Total *n*-6	8.16 ± 0.31	8.36 ± 0.72	26.59 ± 0.34	25.76 ± 0.28
Total *n*-3	1.19 ± 0.04	1.18 ± 0.03	7.92 ± 0.17	7.80 ± 0.21

Values are means ± SD; n=4-9 per group.

Supplementary Table S3. Glucose and insulin dynamics in fasting state and during intravenous glucose and insulin tolerance tests in *rats*

		CTRL n=11-12	MCT n=7-8
PN 42 Fasted	Fasting glucose (mM)	9.84 ± 1.51	8.37 ± 1.14*
	Fasting insulin (pM)	101.42 ± 56.6	107.60 ± 97.21
	HOMA-IR	45.8 ± 28.5	39.7 ± 37.5
PN 84 i.v.GTT	Fasting glucose (mM)	6.86 ± 0.99	7.02 ± 1.70
	Fasting insulin (pM)	341.93 ± 115.19	440.86 ± 203.80
	HOMA-IR	85.4 ± 30.2	88.1 ± 30.9
	iAUC30glucose (mM*30min)	198.6 ± 64.9	150.4 ± 62.5#
	glucose max value (mM)	79.0 ± 28.5	67.8 ± 25.5
	net glucose increase (mM)	72.2 ± 28.1	60.8 ± 24.9
	iAUC30insulin (nM*30 min)	10.95 ± 3.07	10.72 ± 4.88
	insulin max value (nM)	1.94 ± 0.46	2.16 ± 0.77
	net insulin increase (nM)	1.60 ± 0.40	1.72 ± 0.63
PN 90 i.v.ITT	Fasting glucose (mM)	5.59 ± 1.06	6.28 ± 2.15
	Fasting insulin (pM)	406.83 ± 116.82	505.52 ± 197.13
	HOMA-IR	101.8 ± 44.8	138.6 ± 65.4
	glucose min value (mM)	2.75 ± 0.69	2.60 ± 0.95
	net glucose decrease (mM)	-2.83 ± 0.96	-3.62 ± 1.34#
	dAUC60glucose (mM*60 min)	-72.6 ± 49.2	-105.5 ± 73.4
PN 98 Fasted	Fasting glucose (mM)	12.67 ± 2.47	12.02 ± 2.46
	Fasting insulin (pM)	279.75 ± 235.49	251.95 ± 211.89
	HOMA-IR	169.5 ± 155.0	138.2 ± 114.3

The CTRL and MCT diets were fed to the rats from PN day 2-42; WSD from PN day 43-98. Values are means ± SD. #p<0.1; *p<0.01 compared to CTRL. iAUC, incremental area under the curve; dAUC, decremental area under the curve; HOMA-IR, homeostasis model assessment of insulin resistance.
